# A short feature vector for image matching: The Log-Polar Magnitude feature descriptor

**DOI:** 10.1371/journal.pone.0188496

**Published:** 2017-11-30

**Authors:** Damian J. Matuszewski, Anders Hast, Carolina Wählby, Ida-Maria Sintorn

**Affiliations:** 1 Science for Life Laboratory, Uppsala, Sweden; 2 Centre for Image Analysis, Uppsala University, Uppsala, Sweden; 3 Vironova AB, Stockholm, Sweden; University of Campinas, BRAZIL

## Abstract

The choice of an optimal feature detector-descriptor combination for image matching often depends on the application and the image type. In this paper, we propose the Log-Polar Magnitude feature descriptor—a rotation, scale, and illumination invariant descriptor that achieves comparable performance to SIFT on a large variety of image registration problems but with much shorter feature vectors. The descriptor is based on the Log-Polar Transform followed by a Fourier Transform and selection of the magnitude spectrum components. Selecting different frequency components allows optimizing for image patterns specific for a particular application. In addition, by relying only on coordinates of the found features and (optionally) feature sizes our descriptor is completely detector independent. We propose 48- or 56-long feature vectors that potentially can be shortened even further depending on the application. Shorter feature vectors result in better memory usage and faster matching. This combined with the fact that the descriptor does not require a time-consuming feature orientation estimation (the rotation invariance is achieved solely by using the magnitude spectrum of the Log-Polar Transform) makes it particularly attractive to applications with limited hardware capacity. Evaluation is performed on the standard Oxford dataset and two different microscopy datasets; one with fluorescence and one with transmission electron microscopy images. Our method performs better than SURF and comparable to SIFT on the Oxford dataset, and better than SIFT on both microscopy datasets indicating that it is particularly useful in applications with microscopy images.

## Introduction

Local descriptors of features in images have been successfully used in a wide range of applications including object and texture recognition, video and image retrieval, baseline matching, and image stitching [1–6]. The main reasons for their popularity are that they are distinctive, robust to occlusion, and do not require segmentation [4, 6]. Typically the descriptors represent a relatively small region (distinctive feature) in an image with a numerical (or binary) feature vector that is compared with feature vectors obtained from regions in a reference image. The popularity of this feature matching approach has resulted in extensive work to make the descriptors invariant to various image transformations such as translation, rotation, scaling, and illumination [2, 3, 7–10].

Feature-based image matching is typically a four-step process. First, features (characteristic key points) are found with a feature detector in both images. A feature can be described as merely a single point coordinate pair but often contains additional information such as the feature size, shape, and orientation. Second, the features are described using a feature descriptor and coded into numerical feature vectors. Many methods (e.g. SIFT [4] and SURF [11]) combine and cover these two steps in one method. Third, the feature vectors from both images are compared and the corresponding feature set (matches between the images) is found. In the fourth, final step, the feature matches are verified, false positives are removed and the final set of matches is ready for the next step in the application, e.g. image registration.

In the first step, the features are detected and defined by point coordinates and possibly the corresponding neighborhood sizes and orientation, depending on the feature detector method used. It is crucial for a successful matching that the detector finds corresponding features in the two compared images irrespectively of the transformation between them. If there are no corresponding features detected in the two images the matching will fail no matter how good the feature descriptor is. Many scale and affine invariant feature detectors have been proposed [12, 13]. The most popular ones are based on such feature detectors as the Harris corner detection [14], the difference of Gaussians (e.g. SIFT), or the determinant of the Hessian matrix (e.g. SURF). Several papers suggesting different detectors and comparisons between them have been published [2, 3, 12, 15–17]. All these methods have various strengths and weaknesses, and their performance often depends on the application and, more generally, on the transformation type and the distortion degree present in the images [15]. Many feature descriptors are designed to work with certain feature detectors and perform worse when combined with other detectors.

In this paper, we present the Log-Polar Magnitude feature descriptor (LPM) that fits in the second step of the matching pipeline. It computes a feature vector representation of an image feature and is based on the local Log-Polar Transform (LPT) [18] calculated for each feature followed by the Fast Fourier Transform (FFT). The feature vector is obtained by selecting key frequencies in the magnitude spectrum. This selection, or frequency mask, can be adapted to improve the matching performance for different image types. The LPT—FFT combination makes the LPM rotation invariant without any additional normalization step. Consequently, LPM does not require any orientation information from the feature detector which allows using simpler and faster feature detectors without compromising the descriptor (and thus the matching) performance. Moreover, since LPM can work with all feature detectors (all of them provide feature coordinates which is sufficient for LPM) as presented in the Results section it can always benefit from the strengths of the optimal feature detector for a given application.

A similar idea was presented by Kokkinos and Yuille [19], where the input image is first band-pass filtered and then sampled using LPT at multiple scales to obtain phase, amplitude and orientation estimates. Next, the FFT is applied to four different functions constructed from these estimates. Then 32 components from each Fourier image are selected to form a 128-long feature descriptor. Trulls et al. [20] take this idea further by first computing Gaussian derivatives in 8 different directions, and then using the LPT-FFT approach over relatively large image areas (up to a radius of 230 pixels [21]). The size of the resulting descriptor is very large: 3328. Both methods produce much longer feature vectors than the method we propose here and they also require extensive preprocessing and a computationally demanding scale handling. The proposed descriptor was tested in two configurations producing 48- and 56-long feature vectors, respectively. Depending on the application, the feature vectors can be shortened even further.

The method presented here does not perform any pre-computing other than Gaussian smoothing (*σ*^2^ = 1) of the input image, as presented in [18, 22]. There, the feature vector is built using the full local feature LPT-FFT magnitude spectrum. One of the main contributions in this paper is the way the magnitude spectrum of the log-polar sampled image feature is used to build feature vectors, which results in both much shorter feature vectors and higher accuracy. Furthermore, we show that invariance to image scaling is improved by incorporating the feature size information from the feature detector, if a feature detector providing such information is used. We also show that LPM can work with various feature detectors. Finally, we present and discuss a comparison with the popular descriptors SIFT and SURF on three image datasets with various transformation challenges.

MATLAB code for LPM, its VL Benchmark version as well as the two microscopy datasets used in the method evaluation are freely available at: http://cb.uu.se/~damian/LPM.html. The Oxford image dataset used in the VL Benchmark is publicly available [23].

## Methods

### Log Polar Magnitude feature descriptor

**Fig 1:**
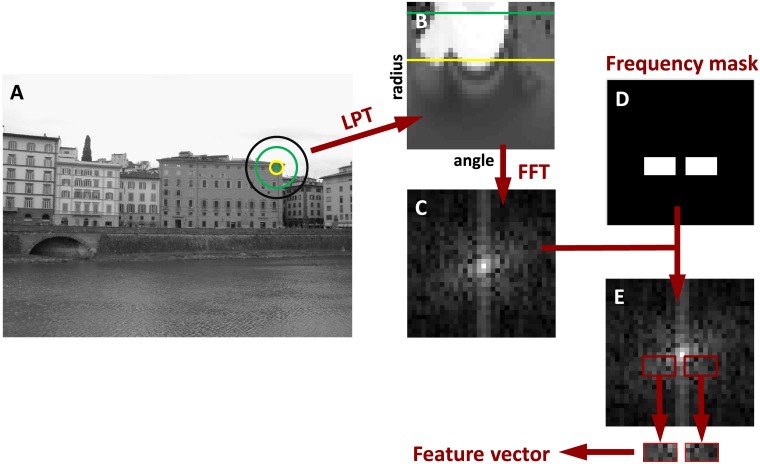
Flowchart of the Log-Polar Magnitude feature descriptor. Each feature, defined by a center (green cross) and a radius (black circle) in the original image (A, image provided by Anders Hast), is Log-Polar Transformed (LPT) to its square representation (B). Next, Fast Fourier Transform (FFT) is used to compute the magnitude spectrum (C, log-scaled image) present in the LPT image. Finally, a frequency mask (D) is used to select the frequencies that compose the feature vector (E). The green and yellow circles in A were added as a reference and correspond to the green and yellow lines in B. The smallest sampling ring, i.e. the one closest to the feature center (marked with the green cross in A), constitutes the bottom row in B whereas the largest ring (marked with the black circle in A) corresponds to the top row in B.

Fig 1 presents a flowchart of the main idea of LPM. Once features have been detected, LPT transforms each disc-shaped neighborhood into a square image [18, 24]. For a feature point with coordinates *x*_0_ and *y*_0_ the LPT resampling is given by

$$\{\begin{array}{c} x=\rho \;\text{cos}\theta \;+x_{0}\\ y=\rho \;\text{sin}\theta \;+y_{0}, \end{array}$$ (1)

where *ρ* is logarithmically distributed in the range [1, *R*], where *R* is the feature radius (in pixels), and *θ* is linearly distributed in the range [0, 2*π*). The values of *ρ* and *θ* correspond to the radial and angular resampling resolution. The LPT resampling resolution is set to 16 or 32 logarithmically distributed rings and 16 or 32 points per ring, respectively, to obtain a square LPT image. In the rest of this paper, these two sampling resolutions are referred to as the 16 x 16 and 32 x 32 sampling strategies. Square sizes of the power of two were used for the LPT representation due to the simpler FFT implementation; however, other dimensions could also be used. The sub-pixel sampling is calculated using Gaussian interpolation with a 5x5 pixel mask.

Circular features do not necessarily require finding their main directions (i.e. orientations) for sampling in rotated squares as it is done in the cases of SIFT and SURF. The LPT is used for representing the circular features as a square image, and any feature rotation is, hence, represented as a 1-D translation in the polar image. Finally, the square representation allows using the Fourier Transform for feature content analysis. The logarithmic sampling of the polar transform is added to make the descriptor less sensitive to erroneous feature size estimation by emphasizing pixels closer to the feature center. It also leads to the descriptor being scale invariant. In contrary to [25] we do not use gradient information explicitly but incorporate the high-frequency information (e.g. edges) when extracting the descriptor from the local Fourier image.

In the next step, FFT [26] is used to compute the frequency domain representation of the LPT image. Then the magnitude is calculated and the frequency components that will form the feature vector are selected. Fig 1D presents the mask corresponding to the selected frequencies. This mask was designed based on the following observations: First, the FFT image is symmetric with respect to its center (constant frequency component); hence, half of the values can be rejected without any information loss. Second, most of the information in an image is usually contained in the low frequencies (visible in Fig 1C as high intensities close to the image center). This is commonly used in image compression, where high frequencies are suppressed to save storage space. Third, the bright vertical line pattern in the frequency image center corresponds to the large intensity difference between the first and the last row of the LPT image. The Fourier Transform assumes that the analyzed signal is periodical. Hence, such a sudden discontinuity in the image border pixel intensities causes artifacts, i.e. high-frequency magnitudes corresponding to the horizontal patterns. Therefore, the frequency mask excludes all primarily horizontal frequencies. Due to the (log) polar sampling, the LPT image is continuous horizontally and hence the same does not hold i.e. there are no artificial high frequencies related to the discontinuity between left and right border pixel intensities. Finally, the rectangular mask presented in Fig 1D gives more priority to the radial than to the angular frequencies, i.e. intensity changes (or patterns) in the direction from the feature center to the outer ring are considered more important (i.e. coded by more frequency components) than the intensity changes along the sampling rings. The final feature vector is composed of the selected frequency magnitudes and normalized to unit length.

Using the LPT—FFT combination gives LPM very interesting properties; any image feature orientation change is represented as an image shift in the LPT which in turn is represented as a phase difference in FFT. Hence, by using the magnitude of the LPT representation in the frequency domain we filter out the orientation variation and keep only information about the frequencies composing the pattern. In other words, the combination of the LPT and the FFT magnitude makes LPM rotation invariant without an additional normalization step. Moreover, LPM does not require any orientation information from the feature detector which allows using much simpler and faster detectors without compromising the descriptor performance.

Note that there is one important fact connected to LPT sampling and its influence on the frequency spectrum. If the same spatial sampling frequency (i.e. the same arc length distance between sampling points on all rings) is to be kept, a different number of sampling points on each ring (smaller on the inner rings and larger on the outer ones) must be used and consequently a log-polar image that is triangular rather than square is obtained. By keeping the number of sampling points constant in each ring the outer rings are in fact subsampled. This may result in higher intensity differences between these points. The last rings will thus contribute more to the high frequencies. The proposed frequency mask, however, is designed to only keep the low-frequency contribution. Hence, LPM focuses more on the fine details around the feature center and the smooth intensity changes closer to the feature border.

#### Parameter selection

**Fig 2:**
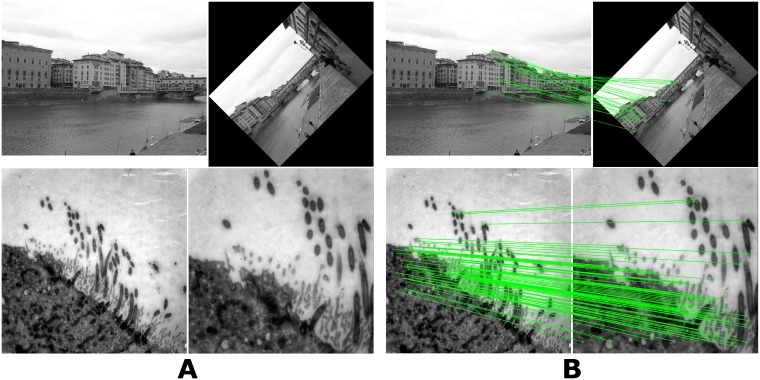
Sample images used for training the general purpose frequency mask and feature scaling coefficients of the Log-Polar Magnitude feature transform (LPM)(A) and the corresponding results (B). The first row presents two natural scene images captured in Firenze, Italy by Anders Hast. The top-right image was additionally artificially rotated (by 45 degrees) and scaled (factor 0.65 with bicubic interpolation). The second row shows two transmission electron microscopy (TEM) images of a cell section with transversely cut cilia (hair-like cell protrusions) acquired at two different magnifications.

In order to produce a frequency mask corresponding to a general purpose feature descriptor generating short feature vectors, we used two pairs of natural scenes and two pairs of transmission electron microscopy (TEM) images during the LPM training. Fig 2 presents one image pair of each type in the training dataset. Each pair poses a different transformation problem. The natural scene images contain translation and perspective changes in addition to an artificially added rotation (by 45 degrees) and a scaling (factor 0.65 with bi-cubic interpolation). The TEM images were acquired at different magnification levels and with small spatial offsets which lead not only to a change in scale and translation but also to illumination and noise level differences related to TEM acquisition properties. The resulting frequency mask, therefore, constitutes what we believe is a versatile compromise between different image types, various image transformations, and feature vector length. We have tested several different frequency masks for each sampling strategy and observed that better performance can be obtained when using customized masks dedicated to a specific image type and application (see the S1 File for different frequency mask results on the training set). The final masks consist of double rectangles with 4 x 6 and 4 x 7 frequencies for the 16 x 16 and 32 x 32 sampling strategies respectively, which correspond to floating point feature vectors of lengths 48 and 56. For comparison, SIFT produces feature vectors with 128 values, and SURF—64 or 128.

While the original Harris’ corner detector provides only feature coordinates, both the SURF and the SIFT detectors also return information on the feature scale and orientation. As each of these methods uses different scale spaces these values have to be multiplied by a dedicated feature scaling coefficient *σ* to be represented in pixels. We selected these scale parameter values (one for SIFT and one for SURF detectors) for the training set. We found an optimal *σ* = 14 and 9 for SIFT and SURF features respectively. In the case of the Harris corner detector the feature radius was set to a fixed value of 32 pixels which creates features large enough to be described efficaciously in most cases; however, other radii could also be used.

In all the experiments described here, the same frequency mask and feature scaling coefficient were used in LPM for all image pairs.

### Evaluation

LPM was compared with SIFT and SURF on three evaluation image datasets; the Oxford dataset introduced in [23], a fluorescence microscopy dataset, and a TEM dataset. Both SIFT and SURF are well investigated in the literature and are the most commonly used reference methods for descriptor comparisons. Many improvements and expansions have been proposed for SIFT (e.g. log-polar sampling (sGLOH) [27], and domain size pooling (DSP-SIFT) [7]); some of them can possibly be also applied to LPM. Nevertheless, the original SIFT, thanks to its stability and established popularity, is still widely used in many applications such as texture and material recognition [28] and image retrieval [29, 30]. Therefore, we base our evaluation on the comparison with these two methods, even though more recent feature descriptors such as ORB [10], BRISK [31], LIOP [32], LDB [33], KAZE [34], MROGH [35], and FRIF [36], have been published and are gaining popularity.

The evaluation on the Oxford dataset was performed in two ways. First, we present the results from the VL benchmark tests [37], which implements the descriptor matching score introduced in [15]. In this framework, a match between two feature vectors is found based on the nearest neighbor approach with the Euclidean distance between vectors in the descriptor space as a metric. This ensures that each feature in the transformed image has exactly one match in the reference image. The match verification is performed using a known homography matrix between the two images and the overlap error of the two features. The overlap error is defined as:

$$1-\;\frac{F_{A}\cap F_{H^{T}BH}}{F_{A}\cup F_{H^{T}BH}}<\epsilon_{0},$$ (2)

where *F*_*A*_ is the feature in reference image *A*, *F*_*H*^*T*^*BH*_ is the feature in image *B* transformed with the homography matrix *H*, and *F*_*A*_ ∩ *F*_*H*^*T*^*BH*_ and *F*_*A*_ ∪ *F*_*H*^*T*^*BH*_ are the intersection and union areas between the two features (measured in pixels) respectively. The threshold *ϵ*_0_ (by default set to 0.4) is used to distinguish between a true match and an accidental feature overlap. It takes values between 0 and 1; the larger *ϵ*_0_ the more tolerant is the feature verification and the more matches are considered correct. Finally, the number of correct matches is defined as the intersection between the descriptor matches and the overlap error matches (the ground truth), whereas the matching score is defined as the ratio between the number of correct matches and the smaller number of the features between the two images that are fully contained (visible) in both reference *A* and the transformed image *B*:

$$matching\;score=\frac{\left|correct\;matches\right|}{\text{min}(|features_{A}|,|features_{B}|)\;},$$ (3)

where *features*_*A*_ and *features*_*B*_ are the features in image *A* and *B*, respectively, that are fully visible in both.

The overlap error that constitutes the basis for this evaluation framework has several problems that are discussed in the original paper [15] and further criticized in [38]. First, feature scaling has a large influence on their overlap and hence the identification of correct matches. In order to overcome this problem, the authors of the original paper suggest normalizing the feature scales before computing the overlap error. However, as noted by the authors, this procedure may make the evaluation results deviate from the performance in a real application where the true feature scales are usually not known and it is therefore not recommended in practical applications. Second, the overlap error is strongly influenced by the feature density; it favors dense, redundant and overlapped feature detections. In practice, if the number of features becomes very large, the image might get so cluttered with features that some of them may be matched by accident rather than by design, which makes the matching verification unreliable in such a case. Considering the fact that the detectors typically find large numbers of densely clustered features in image areas with a lot of texture, this constitutes a large problem in evaluating the descriptors in many applications. Finally, this framework assumes that each feature has at most one correct match. This, however, may not be true if the scale difference between the two images is large. In such a case true one-to-many matches are possible.

Considering all these problems we decided to design an alternative evaluation approach. In this approach, matches between feature vectors are found with a threshold-based method implemented in MATLAB. The threshold value represents a percentage of the distance from a perfect match defined as the full overlap of two feature vectors normalized to unit lengths. Two feature vectors are considered a match when the distance between them is less than the threshold (set to 1%). We used the sum of squared differences between the corresponding feature vector values for the distance metric and an exhaustive search in order to assure that all potential matches are found. Using the normalized (percentage) distance allows for an unbiased descriptor comparison despite the difference in their feature vector lengths. We also used the Lowe condition introduced in [4] that filters out ambiguous matches based on the distance ratio between the 2^*nd*^ nearest and the nearest neighbor as:

$$\frac{distance\;to\;the\;2nd\;nearest\;neighbor}{distance\;to\;the\;1st\;nearest\;neighbor}<\tau_{L},$$ (4)

where *τ*_*L*_ is the Lowe threshold set to the default 0.6 [4]. This condition requires correctly matched points to be substantially closer to each other than to any other feature vector and provides much more reliable matching than a simple threshold-based approach. The second-closest match can be understood as a density estimate of false matches within this portion of the feature space. A pair of points in the feature space has to fulfill both conditions to be considered a match. Finally, one-to-many matches were allowed in order to handle cases with large scale differences.

**Fig 3:**
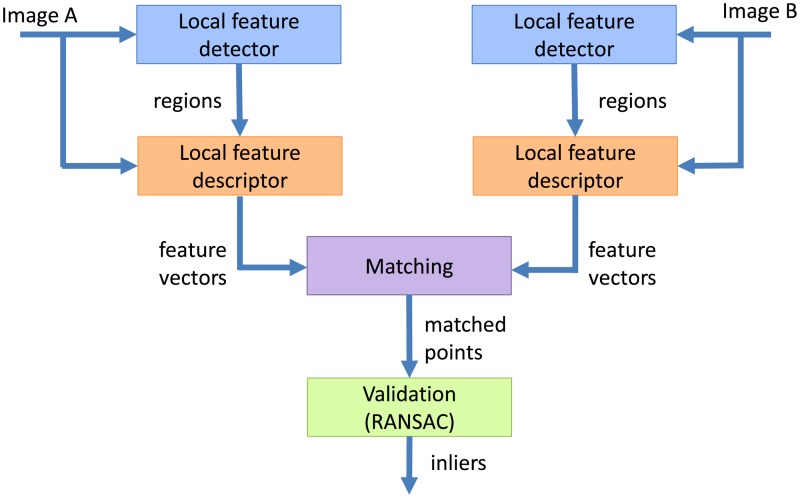
The proposed feature descriptor evaluation approach.

To verify which of the matches are correct we used the Optimal RANSAC [39]. It is an iterative re-estimation RANSAC version using local optimization capable of finding the optimal feature matching subset in heavily contaminated sets. This is possible even when the correct matches constitute less than 5% of all matches, which combined with its repeatability make the Optimal RANSAC an effective tool for verification of correct matches. Fig 3 presents the data flowchart in the proposed evaluation approach. LPM is used in the local feature descriptor block.

In this evaluation approach, the performance of each of the algorithm blocks (local feature detector, descriptor, matching, and validation) can be measured by keeping all the other blocks fixed. In our descriptor evaluation, we used the same local feature detectors for all descriptors and kept both matching thresholds constant and equal to their default values for all image pairs. The performance can be measured in the number of correct matches (inliers) and the inlier ratio:

$$inlier\;ratio=\frac{\left|inliers\right|}{\left|all\;matches\;found\right|}.$$ (5)

## Results

The performance on the Oxford dataset was evaluated using both the VL benchmark evaluation framework and our suggested evaluation approach. Due to the drawbacks of the VL benchmark evaluation approach described in the Evaluation section, the performance on the microscopy datasets was only evaluated using our suggested alternative evaluation approach.

### Oxford dataset

The Oxford image collection has become the standard benchmark dataset for both local feature detectors and descriptors [7–10, 13]. It contains natural scene image subsets with different transformations of gradually increasing strength. Each subset focuses on a particular transformation: blurring, illumination, JPEG compression, viewpoint, and scaling plus rotation.

In all the tests with the Oxford dataset, the SIFT feature detector was used to find local features in the images. By using exactly the same feature set for all descriptors their performance could be reliably compared.

**Fig 4:**
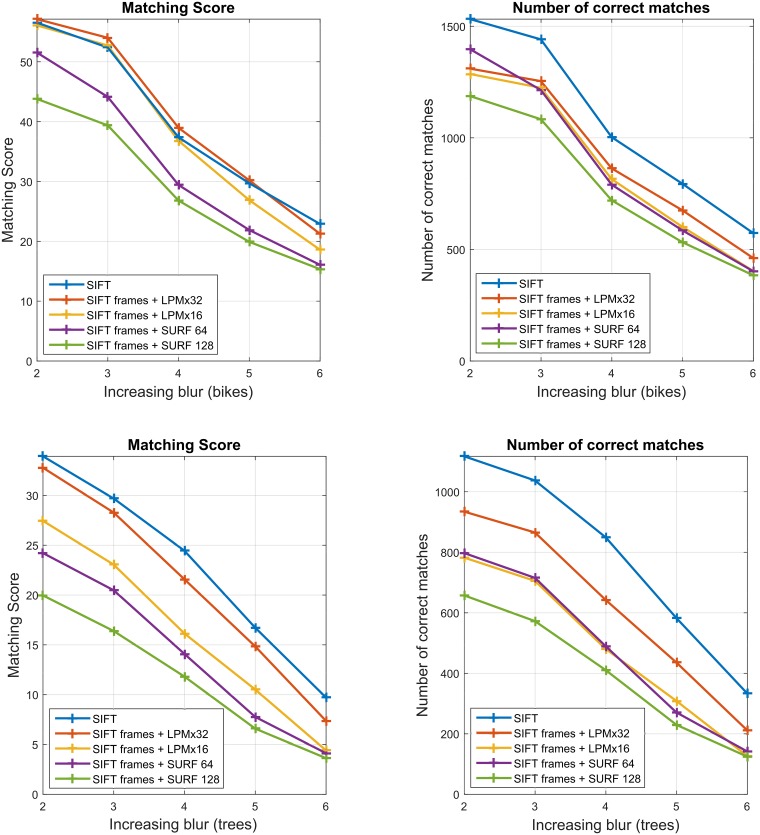
The VL benchmark performance comparison of the three descriptors. SIFT, LPM (in the two sampling variants: 16 x 16 and 32 x 32 resulting in 48- and 56-long feature vectors respectively), and SURF (with the two feature vector lengths: 64 and 128). The same feature detector (SIFT) was used with all descriptors. The plots present the results for the two image subsets with different levels of blur in the Oxford dataset: *bikes* (upper row) and *trees* (lower row).

Fig 4 presents the VL benchmark results for the two image subsets with different blur levels. In both cases, LPM with the 32 x 32 sampling achieves comparable matching scores to SIFT, despite much shorter feature vectors (56 and 128, respectively). As expected, LPM with the lower sampling (16 x 16) performs slightly worse. This is true for all the other image subsets and is related to the amount of image information described in the feature vector.

It can also be observed that for all descriptors the number of correct matches and matching score curves are correlated but at the same time they do not have exactly the same slope and the same order. This is due to the fact that the features located too close to the image borders are removed by the LPM descriptor (due to feature rescaling and sampling interpolation), which changes the denominator in the matching score formula (3). Moreover, the number of features detected in the transformed images varies with the degree of transformation, again true for all data subsets (different types of transformations).

Also common for all the image subsets in the Oxford dataset is that SURF with a shorter feature vector (64) outperformed its longer version (128). Longer feature vectors tend to “over-describe” local features which usually results not only in a lower matching score but also in a noticeably lower number of detected matches. Shorter feature vectors are more forgiving during the matching which increases the descriptor invariance to image transformations but at the same time increases the false positives rate.

**Fig 5:**
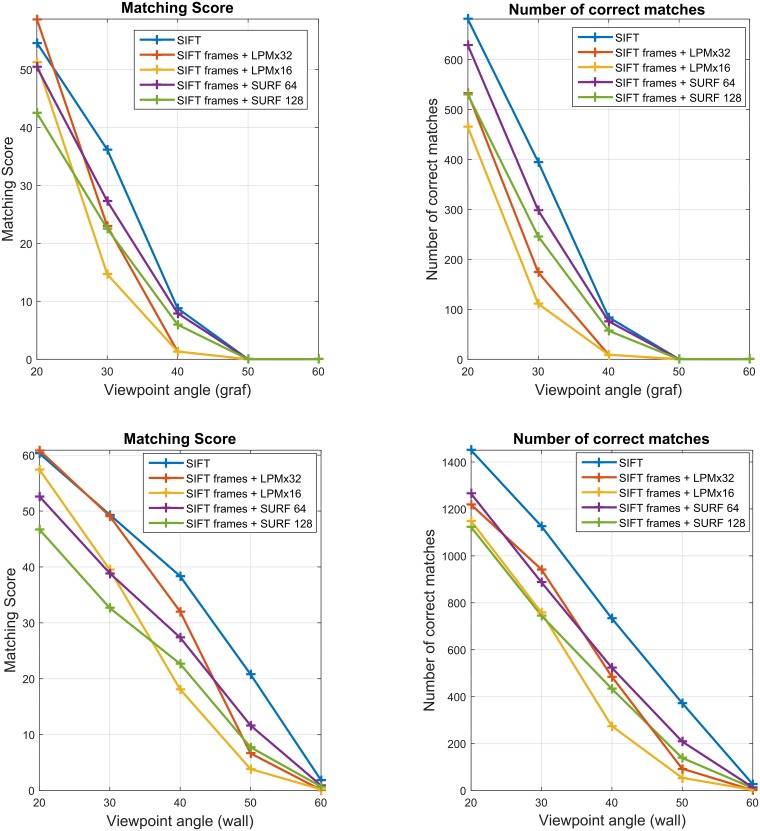
The VL benchmark performance comparison of the three descriptors. SIFT, LPM in the two sampling variants: 16 x 16 and 32 x 32, resulting in 48- and 56-long feature vectors respectively, and SURF with the two feature vector lengths: 64 and 128. The same feature detector (SIFT) was used with all descriptors. The plots present results for the two image subsets with increasing viewpoint angles in the Oxford dataset: *graf* (upper row) and *wall* (lower row).

Fig 5 presents the descriptors performance comparison on two image subsets with various changes in viewpoint angle. We can observe that both the number of correct matches and the matching score decrease drastically with the viewpoint angle increase. Since all the descriptors are based on square or circular features they are not able to adapt to the deformed shapes caused by this transformation. In order to improve the performance in this kind of problems, the features would have to take rectangular or elliptic shapes with their additional orientation and elongation estimation. While not suitable for large viewpoint angle changes LPM proved in these two examples to be still sufficient and as effective as SIFT when the angle difference is less than approximately 25 degrees.

**Fig 6:**
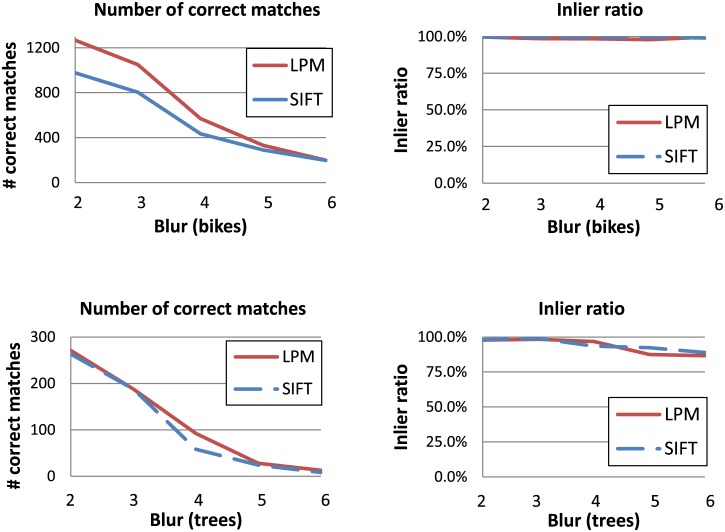
Performance comparison between SIFT and LPM with the 32 x 32 sampling and 56-long feature vectors. The same feature detector (SIFT) was used with both descriptors. The plots present the results of the alternative (threshold- and RANSAC-based) evaluation framework for the two image subsets with an increasing blur in the Oxford dataset: *bikes* (upper row) and *trees* (lower row).

**Fig 7:**
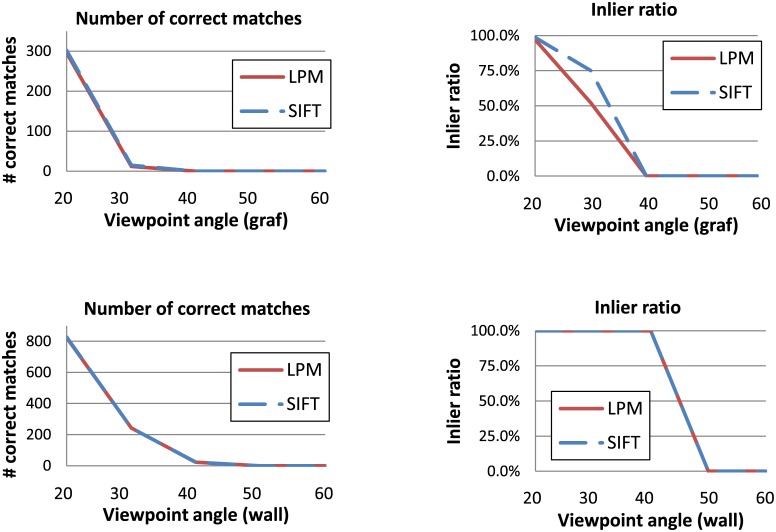
Performance comparison between SIFT and LPM with the 32 x 32 sampling and 56-long feature vectors. The same feature detector (SIFT) was used with both descriptors. The plots present results of the alternative (threshold- and RANSAC-based) evaluation framework for the two image subsets with increasing viewpoint angles in the Oxford dataset: *graf* (upper row) and *wall* (lower row).

Figs 6 and 7 present the results corresponding to Figs 4 and 5 respectively, but with the alternative evaluation framework. Since SURF and LPM with the 16 x 16 sampling typically performed worse than LPM with the finer sampling version on all image pairs in all datasets, only the results for SIFT and LPM with the 32 x 32 sampling are presented. As in the VL benchmark, both descriptors were used with the same features set found with the SIFT detector.

In the case of the image subsets with different blur levels LPM generated more true matches than SIFT (see Fig 6). At the same time, no difference between the two methods can be observed in the inlier ratio. This indicates that LPM describes the features in a more robust way, without compromising the accuracy, i.e. it generates more true feature pairs located very close to each other in the feature space than SIFT. On the other hand, SIFT showed a higher inlier ratio in the second image pair of the *graf* subset (see Fig 7). Nevertheless, there is no noticeable difference between the two descriptors in terms of the number of correct matches. This means that in this case SIFT was more accurate and generated less false matches. In the second viewpoint subset, *wall*, both methods performed similarly.

The results from both evaluations performed on the remaining image subsets from the Oxford dataset (JPEG compression, blur, illumination, viewpoint angle, and scale plus rotation) are published in S1 File. In all cases, LPM with the 32 x 32 sampling and the 56-long feature vector performed better than SURF (both 64- and 128-long) and comparable to SIFT.

### Fluorescence microscopy dataset

**Fig 8:**
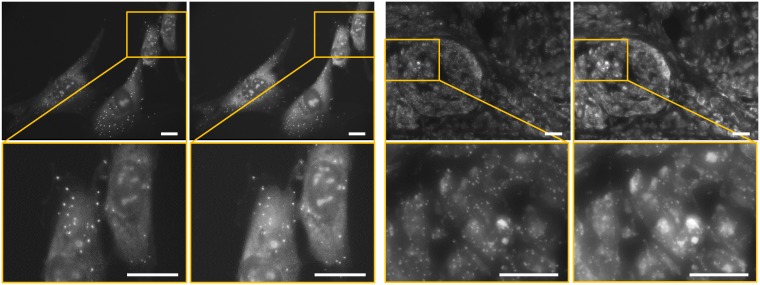
Examples of the fluorescence microscopy images. The two columns to the left show images from the *cell* subset, whereas the two columns to the right present images from the *tissue* subset. The scale bars correspond to 20 *μ*m. The second row shows magnified parts of the images in the first row.

This dataset is composed of two image subsets: one with cultured cells and one with a thin tissue section from a breast cancer tumor obtained from the biobank at the Department of Pathology, Uppsala University Hospital, in accordance with the Swedish Biobank Legislation for a different study as described in [40]. Each subset contains 4 fluorescence microscopy images of the same subject captured at different times, which results in 6 possible image pairs for matching in each case. Fig 8 presents sample images from this dataset. There are several image deviations common for both subsets. First, the fluorescence stain bleaching results in an intensity difference between the images. Second, the images are slightly translated due to the acquisition procedure. Finally, due to biological processes, the position of some of the fluorescent signals (bright dots) is slightly changed between the images. We used this dataset in two experiments; in the first, we compared LPM performance with SIFT, and in the second, we demonstrated the ability of LPM to be used with different feature detectors (SIFT, Harris, and Hessian).

**Fig 9:**
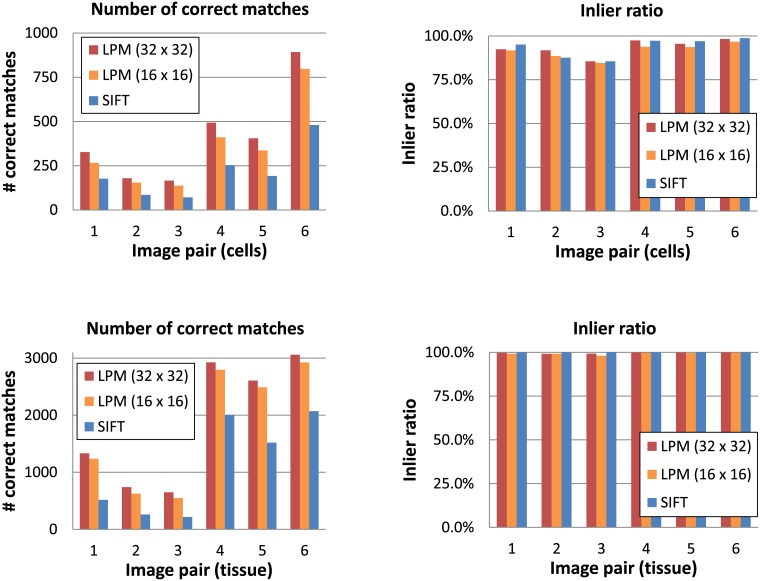
Comparison of the feature matching results obtained with SIFT and the two sampling versions of the Log Polar Magnitude descriptor (LPM) for features found with the SIFT detector. The plots present results for the fluorescence microscopy images: *cells* subset (upper row) and *tissue* subset (lower row).

Fig 9 presents the matching results for SIFT and LPM in the two sampling variants (16 x 16 and 32 x 32) for the fluorescence microscopy dataset. We used our own suggested threshold- and RANSAC-based evaluation approach (see the Evaluation section) and SIFT detector to find features for both descriptors. We can observe that in both subsets and all image pairs, LPM with the 32 x 32 sampling resulted in the largest number of correct matches, followed by LPM with the 16 x 16 sampling and SIFT. There is no substantial difference in the inlier ratio between the descriptors.

**Fig 10:**
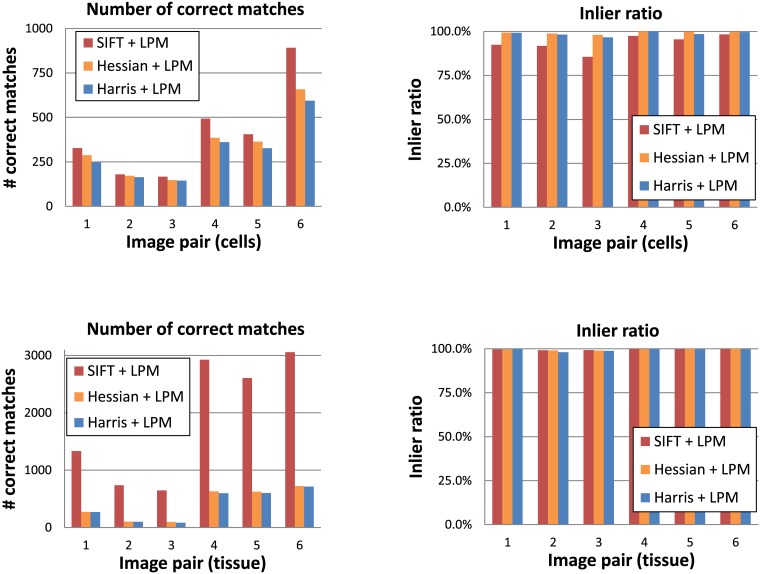
Comparison of different detectors: SIFT, Hessian (blobs) and Harris (corners) in feature matching. In all three cases LPM with the 32 x 32 sampling was used to describe the features. The bar charts present results for the fluorescence microscopy images: *cells* subset (upper row) and *tissue* subset (lower row).

To compare and show how LPM performs using different feature detectors, three common detectors were used: SIFT, Harris (corner detector) [14] and the determinant of the Hessian (blob detector). The matching results are shown in Fig 10. For the Harris and Hessian detectors, only the feature centers were used, whereas their sizes were always fixed to a constant radius of 32 pixels. Since the images in the fluorescence microscopy dataset do not differ in scale the correct feature size detection was not important. We can observe that SIFT was the detector that resulted in the largest number of correct matches in both image subsets. This is caused by SIFT in general detecting many more features than the Hessian or Harris detectors. In fact, such a large number of features is not necessary for a rigid registration and thus it does not constitute a strong SIFT advantage over the other two detectors. Moreover, we can observe that in the case of the cell image subset the optimal feature detector choice is less obvious. In this data subset, both the Hessian and the Harris detectors obtained generally a higher inlier ratio than SIFT. At the same time, they resulted in relatively high numbers of correct matches, which makes them attractive alternatives to SIFT, especially considering that they are much faster.

### Transmission electron microscopy dataset

**Fig 11:**
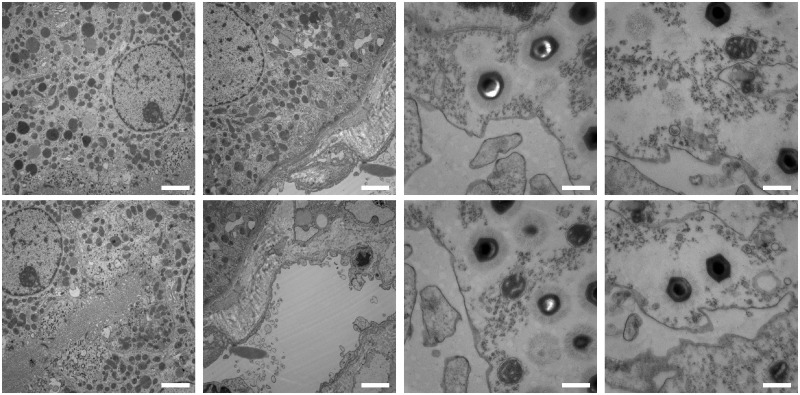
Examples of transmission electron microscopy image pairs organized in columns. The bottom right parts in the images in the first row match the top left parts in the images in the second row. The scale bars correspond to 2 *μ*m. The images in the columns from left to right correspond to the dataset pairs 1, 3, 4 and 5 respectively.

**Fig 12:**
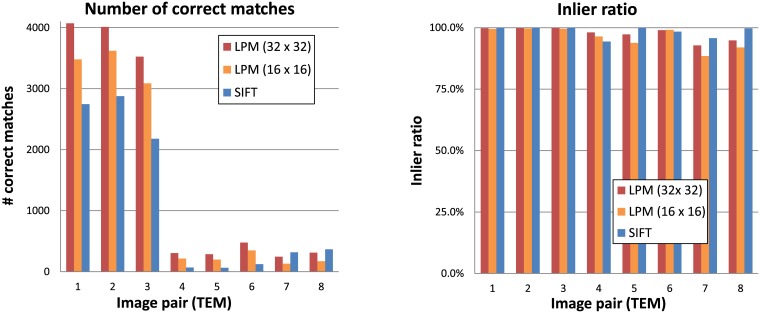
Feature matching results obtained with SIFT and the two Log-Polar Magnitude descriptor (LPM) sampling versions on the features found with the SIFT detector on the transmission electron microscopy image dataset. The first 6 image pairs contained only translation differences whereas the last two image pairs differed in magnification levels.

The transmission electron microscopy dataset is composed of 8 image pairs of tissue sections of cells. The first six (kidney and cells infected with Mimivirus) are translated with respect to each other, whereas the last two (edge of cells with protruding cilia) differ in magnification (scale). Images captured with different magnification are additionally characterized by different noise levels which is typical for TEM acquisition. Fig 11 presents sample images from this dataset. It can be easily observed that the amount of texture varies between the image pairs in this dataset. The first 3 image pairs (of which 1 and 3 are shown in Fig 11) are images with a lot of texture; blob-like structures of various sizes fill the entire images. On the other hand, the last 5 image pairs (see the two image pairs to the right in Fig 11 and bottom row in Fig 2) contain much more regions with relatively little visual information. As a consequence, they constitute a much harder matching problem which is clearly visible in the number of correct matches found in them as presented in Fig 12.

We can observe that LPM for both sampling variants performed better than SIFT on the first 6 image pairs (those with translation only). In the case of the last two image pairs (different magnification), SIFT found more correct matches and achieved higher inlier ratio. The scale difference in these images was too large (zoom factor greater than 2) to be handled by the LPT and scale information from the SIFT feature detector. Nevertheless, LPM with the 32 x 32 sampling was only slightly worse than the SIFT descriptor in the number of correct matches.

## Discussion

Matching of two patterns or objects in images depends on the feature detector performance. The best detector consequently finds the same features despite any image transformation. If two images are a match but the detector provides very different feature sets for each of them the matching will fail, no matter how good the feature descriptor is. Different feature detectors perform differently with various image types. Hence, the ability to work with any detector is a great advantage of the proposed LPM descriptor. Since LPM uses only the center coordinates and (optionally) the radius of a feature it can benefit from many recent detector improvements, e.g., [34], as well as any new feature detectors proposed in the future. Moreover, LPM does not rely on gradient calculations to assure its rotation invariance, as e.g. SIFT, SURF and HOG [41]. Many blob-like features (very typical in microscopy images) have no dominant direction, making LPM suitable for such applications. Furthermore, as the feature orientation does not have to be estimated, much simpler and thus faster feature detectors can be used with LPM without compromising its performance.

Note that the constant component (corresponding to the average pixel intensity in a feature) is not part of the frequency mask composing the feature vector. Therefore, LPM is invariant to uniform illumination changes in the feature areas. Moreover, since the frequency mask selects primarily low frequencies to build the feature vector, LPM is practically indifferent to the frequency-based compression artifacts observed in e.g. JPEG compression (see Fig 1 in S1 File for the LPM evaluation results on the Oxford JPEG compression dataset).

The frequency sampling results in increased robustness to image transformations and a shorter feature vector. The proposed descriptor can be further adapted to work with an application specific image type by customizing the frequency component selection (optimization) in the feature vectors. This gives the potential of adjusting the descriptor to distinguish between very specific types of features and patterns. While constructing the general purpose frequency mask we also evaluated the mask parameters for the different image sets (see S1 File). We observed that when customizing the frequency mask for only the natural scene or TEM image subset the inlier ratio was improved by almost 10% for the image pairs of this type in the training set. The optimal frequency mask for a given application can be found using the observations described in the Methods section or through an exhaustive search of all potential masks of a given size (number of frequency components). Moreover, the feature vector length can be easily adapted to match hardware or application requirements. The proposed method also has the possibility of parallelization and very fast implementation. LPM with *N* x *N* LPT sampling has the computational complexity *O*(*N*^2^*logN*), i.e. the same complexity as its slowest component—FFT (see S1 File for the complexity analysis). Since LPM is based on the Fourier transform and operates on short feature vectors it constitutes a particularly attractive feature descriptor for applications in which image matching is to be implemented on embedded systems or FPGA.

During the experiments, we noticed that the optimal feature scaling coefficient *σ* (for a given detector) varies between image types. Here, *σ* was set to a fixed value that represents a compromise between the TEM training dataset (that had a larger optimal *σ*) and the natural scene training dataset (that had a smaller optimal *σ*) (see S1 File for the experiment results). Hence, *σ* can also be adjusted to improve the performance for a particular image type.

Scale invariance is handled to some extent by the LPT sampling through its logarithmic distribution of the sampling rings, the feature radius (if provided by the feature detector) and *σ*. However, not all feature detectors provide information about their sizes (e.g. Harris corner detector). In our experiments, we observed that small scale changes can be successfully handled by the LPT itself. However, when the scale difference is larger than 2, a feature size estimation becomes necessary. In cases when such information is unavailable, or when the scale difference between images is very large, a scale pyramid can be incorporated into the matching procedure. The scale pyramid can be used to estimate the zoom factor by comparing the number of matched features (before the validation) when one of the images is resized to half or to a quarter of its original dimensions. In particular, we suggest comparing 5 scenarios for input images A and B: A vs B, A vs 0.5 B, A vs 0.25 B, 0.5 A vs B, and 0.25 A vs B. In each scenario, we detect features and compute the feature vectors for the resized images. The final feature vectors set for A and B is then taken from the scenario with the largest matched features number (including the false matches). Of course, the feature center coordinates have to be scaled back to the original image size to compute the homography matrix and to be correctly displayed. This scale pyramid is only needed if substantial scale difference between images is expected, and can be skipped when the scale difference is known a priori, which is typically the case in image stitching, tracking, 3D registration and most microscopy applications. Fig 11 in S1 File presents the benefit of the optional scale pyramid on the TEM dataset. Obviously, when there is no scale difference between images (image pairs 1 to 6) using the scale pyramid gives exactly the same results as not using it. However, when a large scale difference is present between the images, adding the scale pyramid to the descriptor can substantially increase the number of correct matches found with LPM and make it outperform SIFT. However, the same approach could be used to improve SIFT or any other descriptor. Therefore, we did not use the scale pyramid with any of the descriptors in the reported experiments (including those in S1 File).

Our evaluation approach differs from the one proposed in [23]. We believe that the threshold- and RANSAC-based approach is much more intuitive and appropriate since a successful descriptor should be characterized by both a large number of correct matches and a high inlier ratio. A large number of correct matches is important for providing enough information to compute the homography matrix for a given image pair, and a high inlier ratio demonstrates the descriptive power of a descriptor, i.e. its ability to represent features in a distinctive way so that fewer incorrect matches occur. We designed the comparison considering all three descriptors (SIFT, SURF, and LPM) as off-the-shelf tools that work with image pairs with no prior normalization or processing. We decided to keep the matching criterion thresholds constant because in most cases there is no need for tuning this parameter, which is confirmed by a generally high performance of the compared descriptors in all datasets. Moreover, the relative matching threshold allowed for descriptor comparison despite different feature vector lengths.

Finally, it is important to note that the Optimal RANSAC [39] used for match validation, despite being much more stable than the original, may still fail when images have more than one (or non-rigid) transformations due to a perspective or lens distortion. This should be considered during the tests to assure that the results are the same for each run. If the images are camera calibrated and rectified, another type of RANSAC would be preferable to fit the fundamental matrix rather than the Direct Linear Transform [39].

## Conclusions

The Log-Polar Magnitude (LPM) feature descriptor presented here offers a performance comparable to SIFT but with a much shorter feature vector length. This results in better memory usage and a much faster matching, making LPM particularly attractive in applications with hardware limitations. Experiments on three independent datasets show that LPM can successfully handle image registration with a wide range of transformations: translation, rotation, blurring, JPEG compression, variation in illumination and to some extent—scaling and viewpoint changes. We have described and proposed a general feature descriptor based on the training set composed of both natural scene and microscopy images. We have also shown that through a careful selection of frequency components constituting the feature vectors, the descriptor can be adjusted to a particular application problem, specializing it to distinguish between particular patterns and thus further improve its performance in the application domain. Moreover, LPM can work with any feature detector that provides the basic feature information; feature coordinates and (optionally) its size (radius). Since LPM does not require orientation information from the detector, much simpler and faster detectors can be used without compromising the descriptor performance.

## Supporting information

S1 File Extended results. This file contains the Log Polar Magnitude feature descriptor evaluation results (using both approaches discussed in this paper) for all image subsets in the Oxford dataset, including the image transformations not presented in the paper: JPEG compression, illumination changes, and scale plus rotation. Moreover, results from the frequency mask and scaling coefficient *σ* selection are also presented. Finally, it also contains results of using LPM with the scaling pyramid illustrated on the TEM dataset.
(PDF)
