# Supplementary material for: A short feature vector for image matching: The Log-Polar Magnitude feature descriptor
Source: PLoS One. 2017 Nov 30;12(11):e0188496. doi: 10.1371/journal.pone.0188496 (PMC5708636; doi:10.1371/journal.pone.0188496)
Supplement: S1 File — This file contains the Log Polar Magnitude feature descriptor evaluation results (using both approaches discussed in this paper) for all image subsets in the Oxford dataset, including the image transformations not presented in the paper: JPEG compression, illumination changes, and scale plus rotation. Moreover, results from the frequency mask and scaling coefficient σ selection are also presented. Finally, it also contains results of using LPM with the scaling pyramid illustrated on the TEM dataset. (PDF) [file pone.0188496.s001.pdf]

A short feature vector for image matching: the Log Polar Magnitude feature descriptor

Damian J. Matuszewski[1,2,*], Anders Hast[2], Carolina Wählby[1,2], and Ida-Maria Sintorn[1,2,3]

[1]Science for Life Laboratory, Uppsala, Sweden
[2]Centre for Image Analysis, Uppsala University, Uppsala, Sweden
[3] Vironova AB, Stockholm, Sweden
*contact: damian.matuszewski@it.uu.se

Parameter selection

Frequency mask selection

Table 1: Inlier ratio and number of correct matches measured for different frequency masks on the training set. The rectangle mask size corresponds to the dimensions of one of the symmetric rectangles used for building the feature vectors for the 32 x 32 sampling strategy as explained in the main paper. The training set contained two transmission electron microscopy (TEM) image pairs and two natural scene image pairs. The results were obtained using the threshold- and RANSAC- based evaluation method. The row in bold corresponds to the frequency mask used in all other experiments and 32 x 32 sampling strategy.

Rectangle mask size	Feature vector length	TEM #1		TEM #2		Natural scene #1		Natural scene #2	
		Inlier ratio	No. matches	Inlier ratio	No. matches	Inlier ratio	No. matches	Inlier ratio	No. matches
2 x 10	40	0.8085	190	0.9748	155	0.8125	52	0.9483	110
3 x 7	42	0.8612	211	0.9759	162	0.8261	38	0.9612	99
3 x 8	48	0.8678	210	0.9756	160	0.8125	39	0.9615	100
3 x 9	54	0.8714	210	0.9817	161	0.8163	40	0.9608	98
**4 x 7**	**56**	**0.8755**	**211**	**0.9824**	**167**	**0.8684**	**33**	**0.9634**	**79**
2 x 10	60	0.8755	211	0.9816	160	0.8367	41	0.9495	94
4 x 8	64	0.9824	211	0.9824	167	0.8649	32	0.8753	79
5 x 7	70	0.8870	212	0.9825	168	0.9333	28	0.9683	61
4 x 9	72	0.8755	211	0.9825	168	0.8684	33	0.9747	77
4 x 10	80	0.8792	211	0.9825	168	0.8649	32	0.9747	77
5 x 8	80	0.8875	213	0.9826	169	0.9310	27	0.9683	61
5 x 9	90	0.8875	213	0.9826	169	0.9333	28	0.9677	60
5 x 10	100	0.8908	212	0.9826	169	0.9286	26	0.9831	58
6 x 10	120	0.9021	212	0.9884	171	0.9600	24	0.9796	48

Table 2: Inlier ratio and number of correct matches measured for different frequency masks on the training set. The rectangle mask size corresponds to the dimensions of one of the symmetric rectangles used for building the feature vectors for the 16 x 16 sampling strategy as explained in the main paper. The training set contained two transmission electron microscopy (TEM) image pairs and two natural scene image pairs. The results were obtained using the threshold- and RANSAC- based evaluation method. The row in bold corresponds to the frequency mask used in all other experiments and 16 x 16 sampling strategy.

Rectangle mask size	Feature vector length	TEM #1		TEM #2		Natural scene #1		Natural scene #2	
		Inlier ratio	No. matches	Inlier ratio	No. matches	Inlier ratio	No. matches	Inlier ratio	No. matches
3 x 4	24	0.6607	111	0.8571	72	0.4630	25	0.7174	66
3 x 5	30	0.6747	112	0.8621	75	0.6410	25	0.7901	64
4 x 4	32	0.6763	117	0.8953	77	0.4651	20	0.7733	58
3 x 6	36	0.6975	113	0.8953	77	0.6410	25	0.8125	65
4 x 5	40	0.7151	123	0.8966	78	0.6286	22	0.8551	59
3 x 7	42	0.7267	117	0.9036	75	0.6744	29	0.8267	62
**4 x 6**	**48**	**0.7305**	**122**	**0.9205**	**81**	**0.7097**	**22**	**0.8333**	**60**
4 x 7	56	0.7500	123	0.9310	81	0.7419	23	0.8462	55
4 x 8	64	0.7593	123	0.9326	83	0.8846	23	0.8438	54

Scaling coefficient selection

Table 3: Inlier ratio and number of correct matches measured for different scaling coefficients for the SIFT detector on the training set. The training set contained two transmission electron microscopy (TEM) image pairs and two natural scene image pairs. The results were obtained using the threshold- and RANSAC- based evaluation method and LPM with the 32 x 32 sampling strategy. The row in bold corresponds to the scaling coefficient used in all other experiments and the SIFT detector.

Scaling coefficient	TEM #1		TEM #2		Natural scene #1		Natural scene #2		Average inlier ratio
	Inlier ratio	No. matches	Inlier ratio	No. matches	Inlier ratio	No. matches	Inlier ratio	No. matches	
1	0.009	4	0.538	179	0.438	7	0.739	17	0.431
2	0.014	9	0.561	207	0.650	13	0.718	28	0.486
3	0.006	4	0.552	233	0.609	14	0.822	37	0.497
4	0.005	5	0.538	287	0.722	26	0.721	49	0.496
5	0.317	230	0.640	373	0.800	32	0.785	62	0.635
6	0.466	280	0.756	472	0.725	29	0.861	62	0.702
7	0.581	323	0.825	529	0.865	32	0.873	62	0.786
8	0.615	356	0.862	593	0.871	27	0.913	63	0.815
9	0.683	384	0.878	618	0.849	28	0.904	66	0.828
10	0.730	402	0.901	657	0.788	26	0.886	62	0.826
11	0.707	399	0.898	668	0.727	24	0.939	62	0.818
12	0.729	412	0.928	692	0.750	24	0.882	60	0.822
13	0.723	418	0.925	702	0.759	22	0.875	56	0.820
**14**	**0.766**	**442**	**0.921**	**722**	**0.826**	**19**	**0.894**	**59**	**0.852**
15	0.774	453	0.917	730	0.765	13	0.898	53	0.839
16	0.771	478	0.912	721	0.900	18	0.926	50	0.877
17	0.774	462	0.926	738	0.500	8	0.842	48	0.761
18	0.767	455	0.929	727	0.857	18	0.891	49	0.861
19	0.753	453	0.908	717	0.529	9	0.875	49	0.766
20	0.762	454	0.900	734	0.714	10	0.854	41	0.807

Evaluation – Oxford dataset

JPEG compression

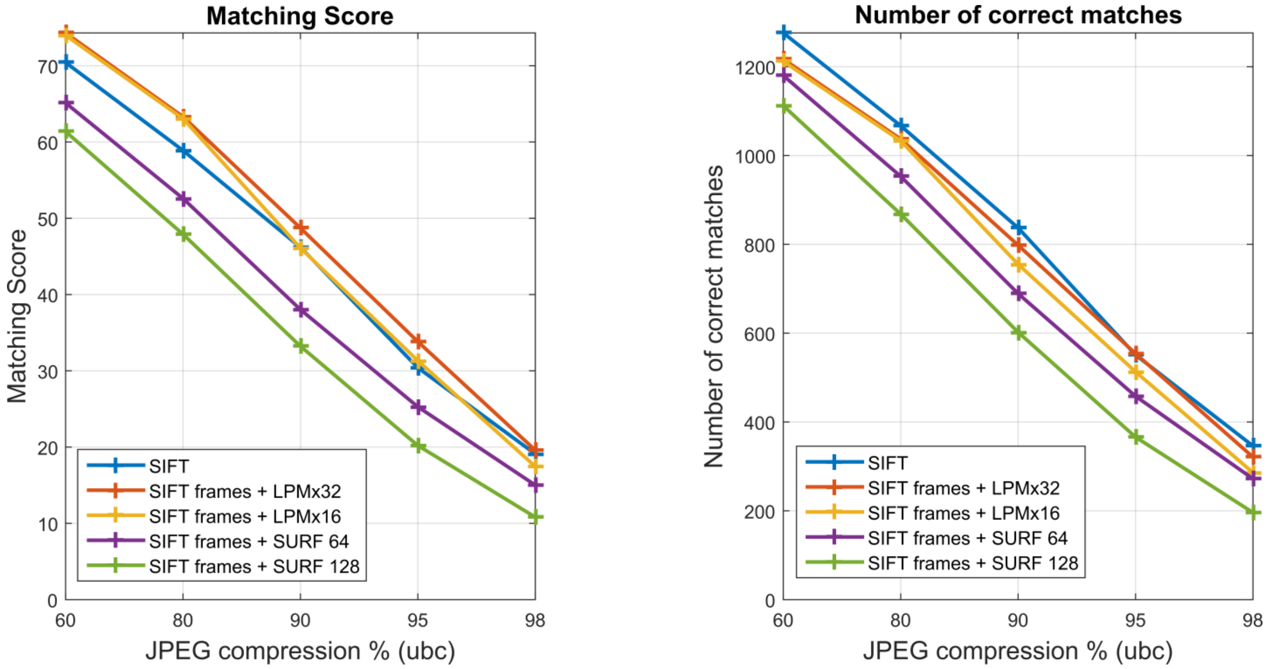

Figure 1: The VL benchmark performance comparison of the three descriptors: SIFT, LPM (in the two sampling variants: 16 x 16 and 32 x 32 resulting in 48- and 56-long feature vectors respectively), and SURF (with the two feature vector lengths: 64 and 128). The same feature detector (SIFT) was used with all descriptors. The plots present the results for the image subset with increasing JPEG compression in the Oxford dataset: *ubc*.

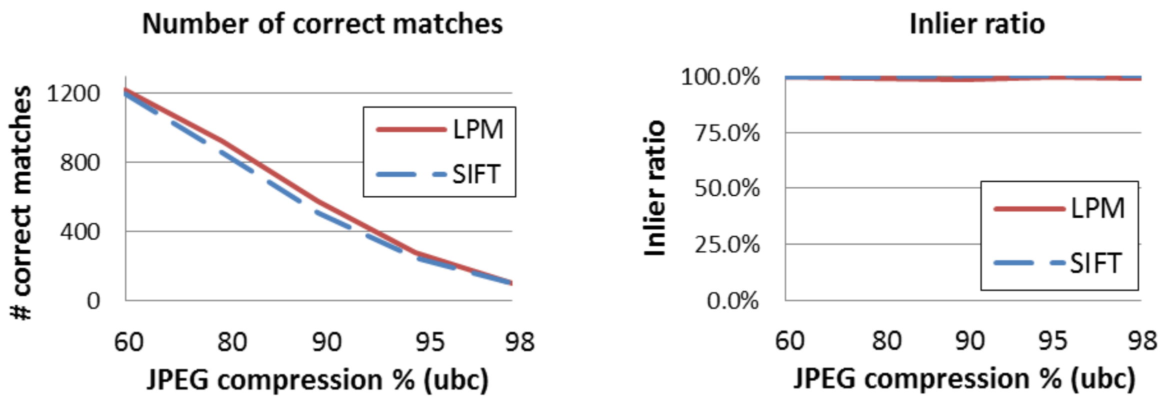

Figure 2: Performance comparison between SIFT and LPM with the 32 x 32 sampling and 56-long feature vectors. The same feature detector (SIFT) was used with both descriptors. The plots present the results of the alternative (threshold- and RANSAC-based) evaluation framework for the image subset with increasing JPEG compression levels in the Oxford dataset: *ubc*.

Blur

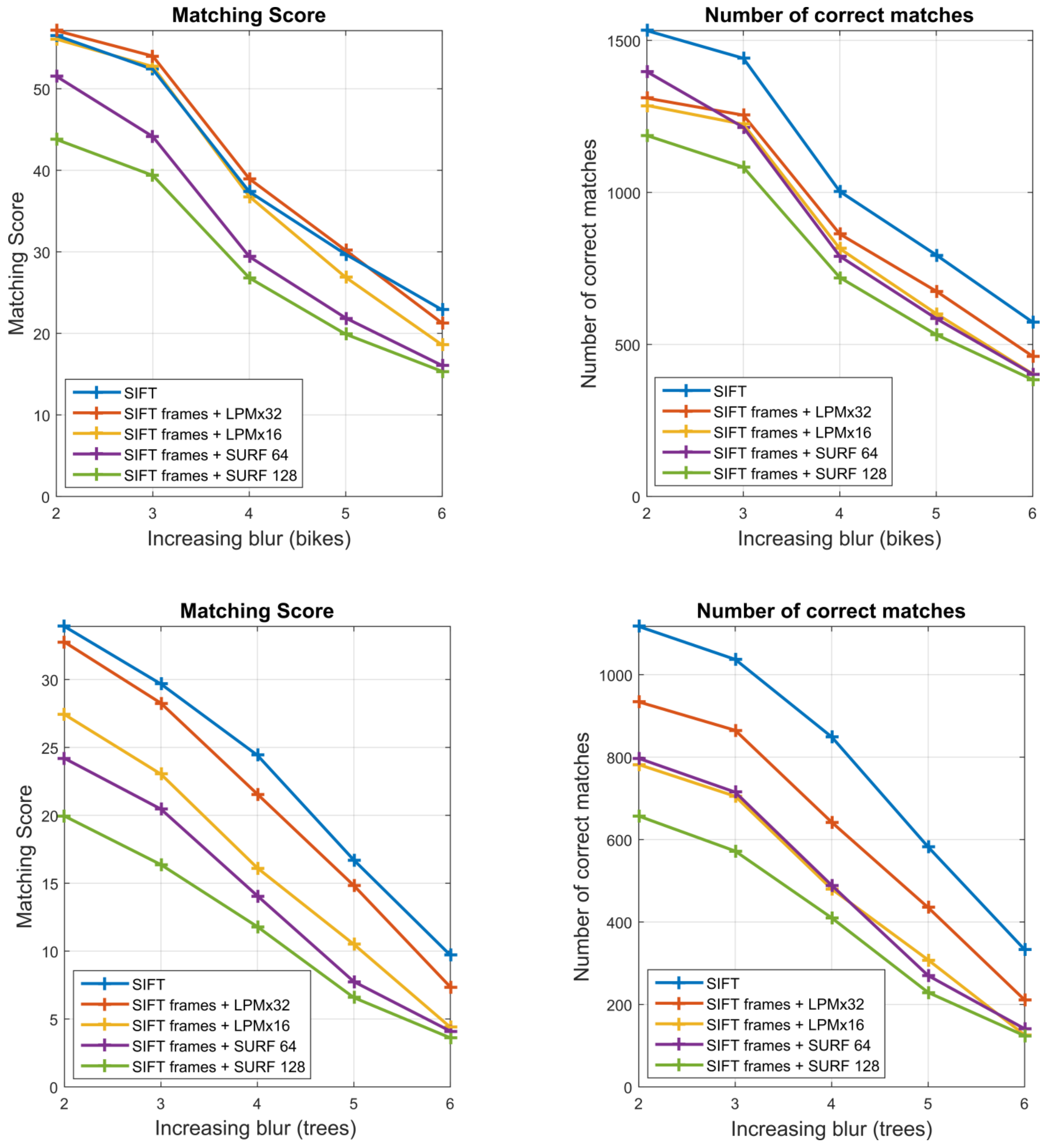

Figure 3: The VL benchmark performance comparison of the three descriptors: SIFT, LPM (in the two sampling variants: 16 x 16 and 32 x 32 resulting in 48- and 56-long feature vectors respectively), and SURF (with the two feature vector lengths: 64 and 128). The same feature detector (SIFT) was used with all descriptors. The plots present results for the two image subsets with different levels of blur in the Oxford dataset: *bikes* (upper row) and *trees* (lower row).

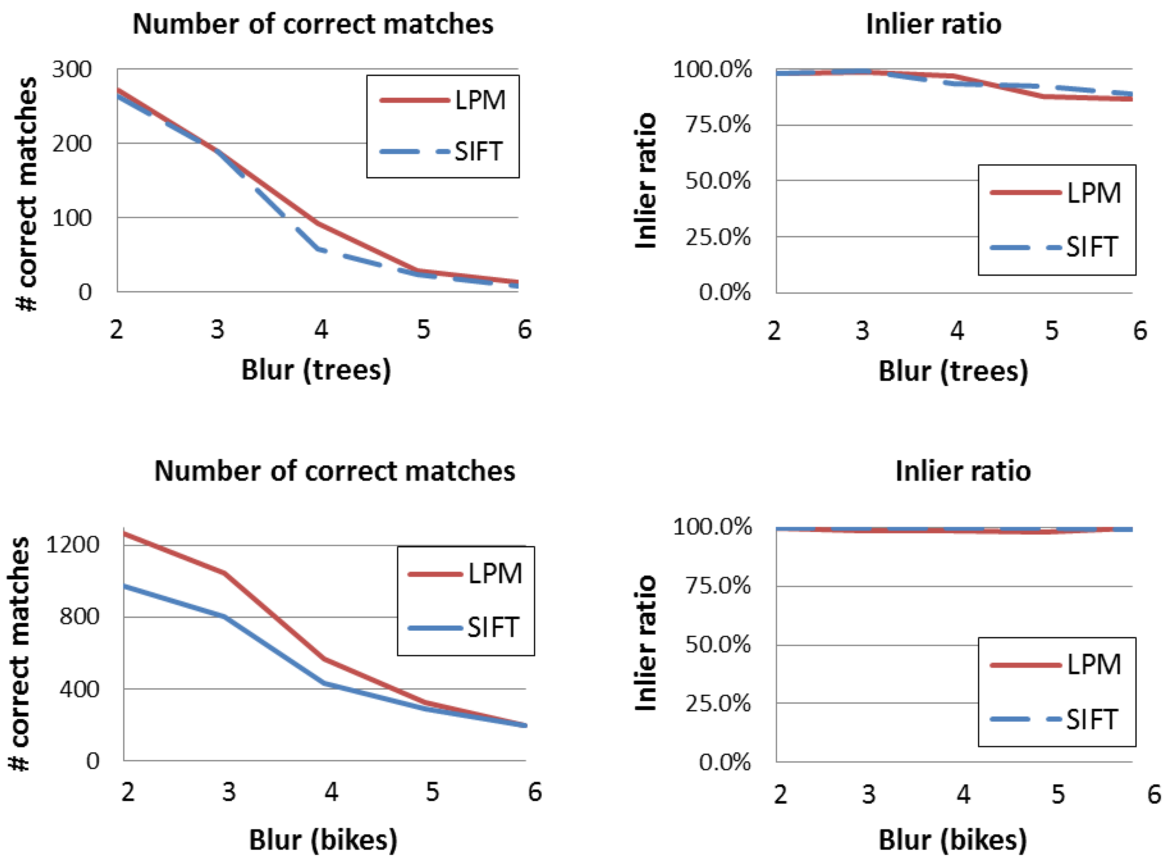

Figure 4: Performance comparison between SIFT and LPM with the 32 x 32 sampling and 56-long feature vectors. The same feature detector (SIFT) was used with both descriptors. The plots present the results of the alternative (threshold- and RANSAC-based) evaluation framework for the two image subsets with increasing blur in the Oxford dataset: *bikes* (upper row) and *trees* (lower row).

Illumination

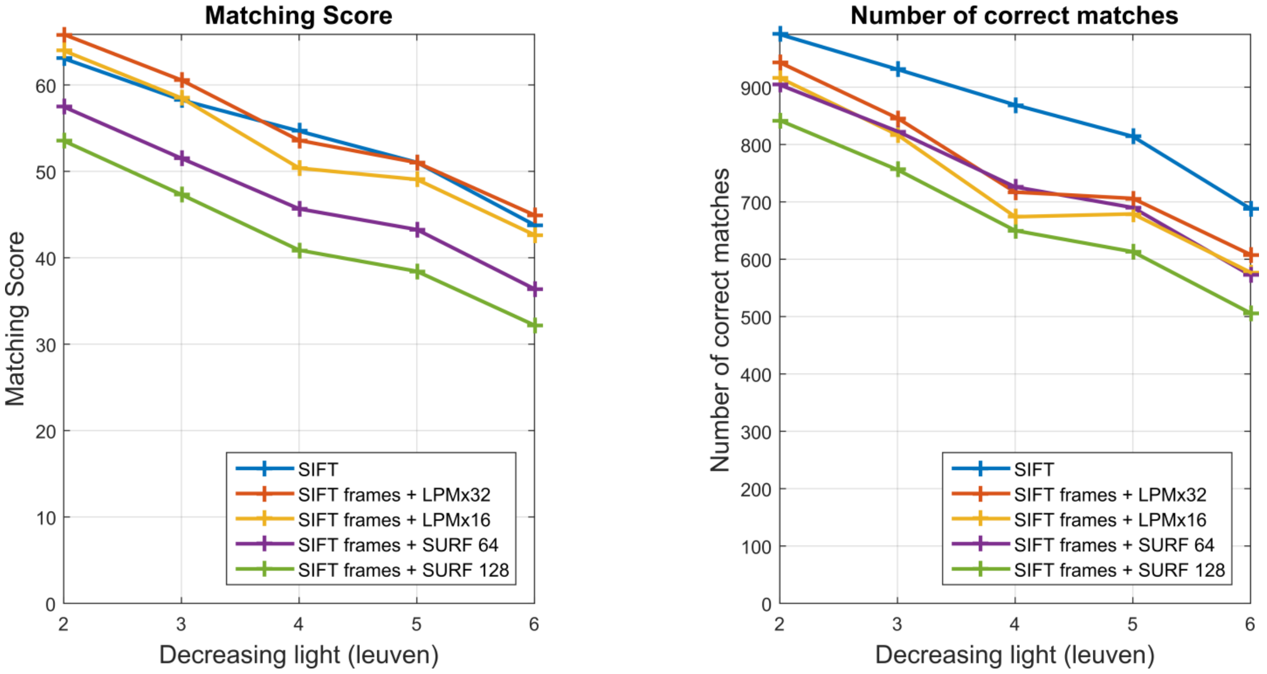

Figure 5: The VL benchmark performance comparison of the three descriptors: SIFT, LPM (in the two sampling variants: 16 x 16 and 32 x 32 resulting in 48- and 56-long feature vectors respectively), and SURF (with the two feature vector lengths: 64 and 128). The same feature detector (SIFT) was used with all descriptors. The plots present results for the image subset with decreasing illumination in the Oxford dataset: *leuven.*

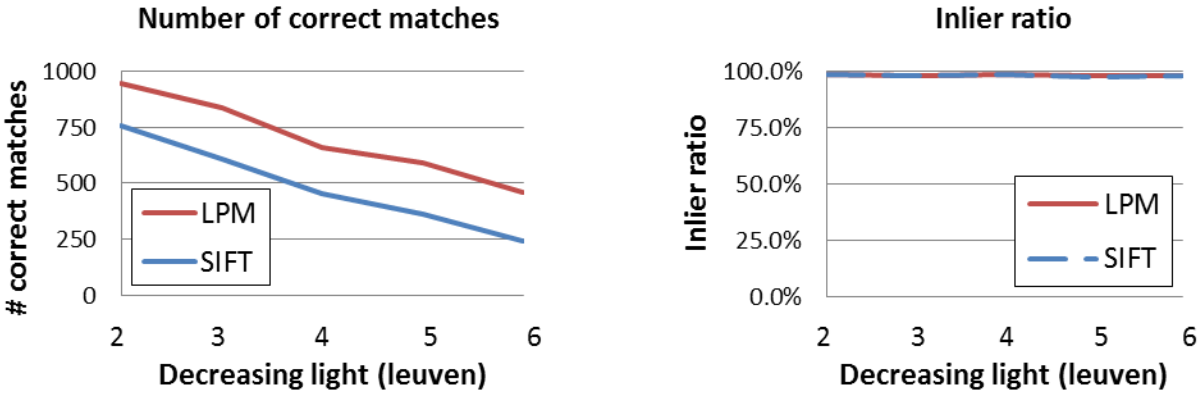

Figure 6: Performance comparison between SIFT and LPM with the 32 x 32 sampling and 56-long feature vectors. The same feature detector (SIFT) was used with both descriptors. The plots present the results of the alternative (threshold- and RANSAC-based) evaluation framework for the image subset with decreasing illumination in the Oxford dataset: *leuven.*

Scale and rotation

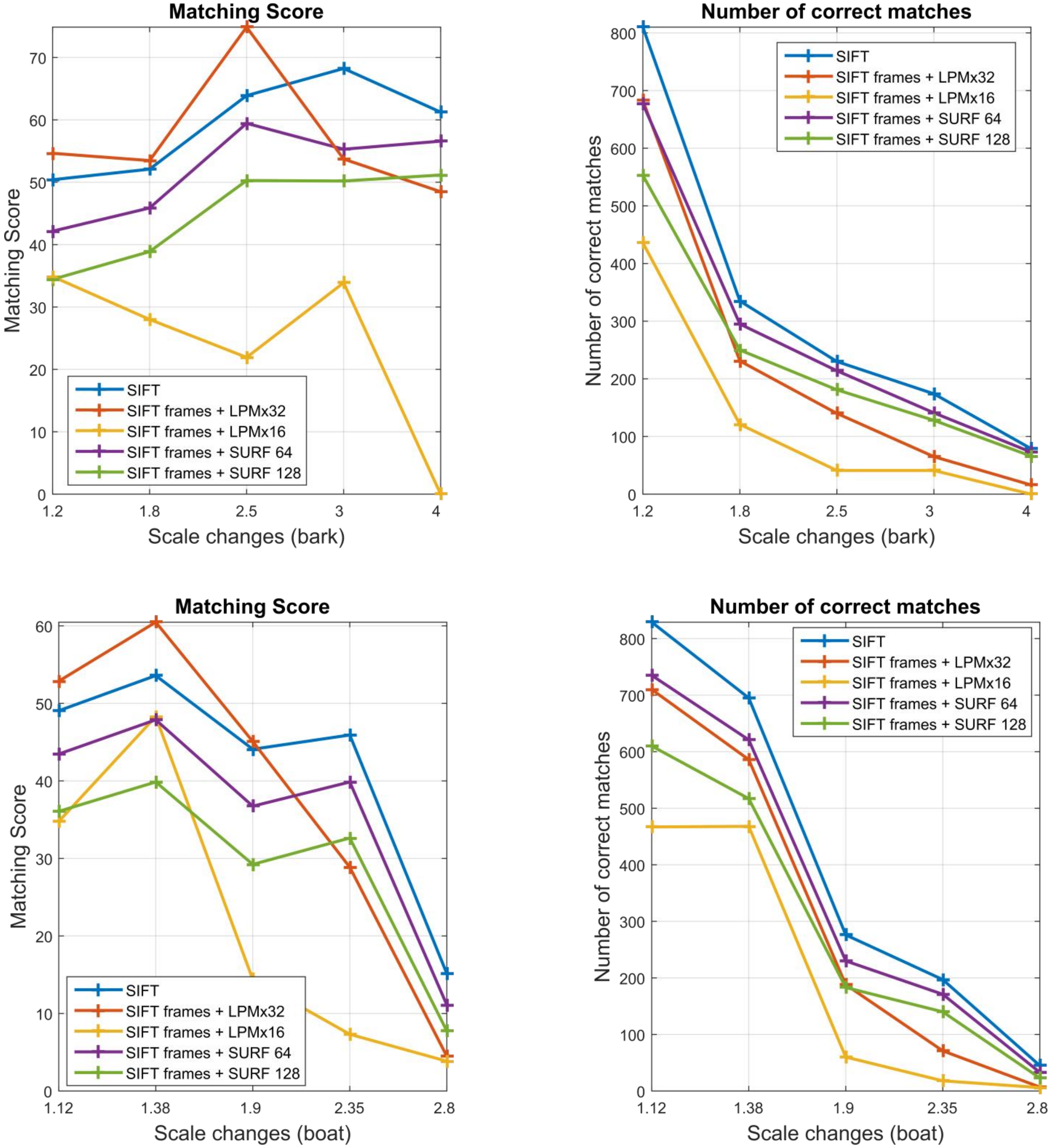

Figure 7: The VL benchmark performance comparison of the three descriptors: SIFT, LPM (in the two sampling variants: 16 x 16 and 32 x 32 resulting in 48- and 56-long feature vectors respectively), and SURF (with the two feature vector lengths: 64 and 128). The same feature detector (SIFT) was used with all descriptors. The plots present results for the two image subsets with different scales and rotations in the Oxford dataset: *bark* (upper row) and *boat* (lower row).

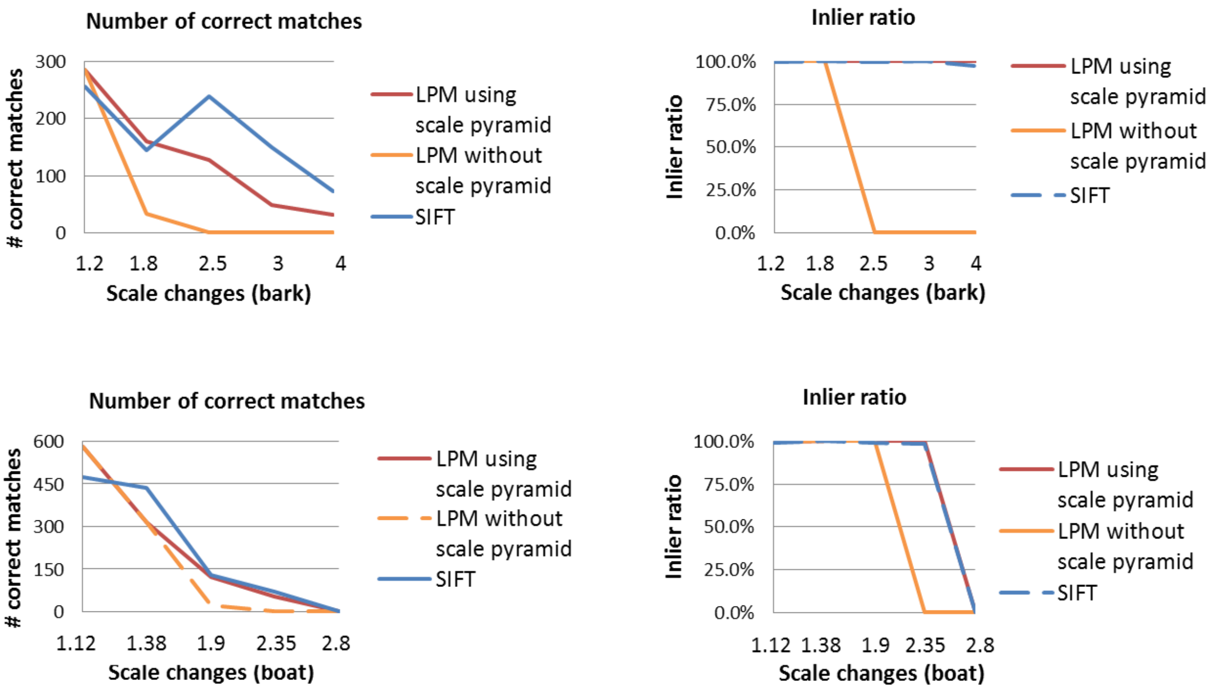

Figure 8: Performance comparison between SIFT and LPM with the 32 x 32 sampling and 56-long feature vectors. The same feature detector (SIFT) was used with both descriptors. The plots present the results of the alternative (threshold- and RANSAC-based) evaluation framework for the two image subsets with scale and rotation changes in the Oxford dataset: *bark* (upper row) and *boat* (lower row).

Viewpoint angle

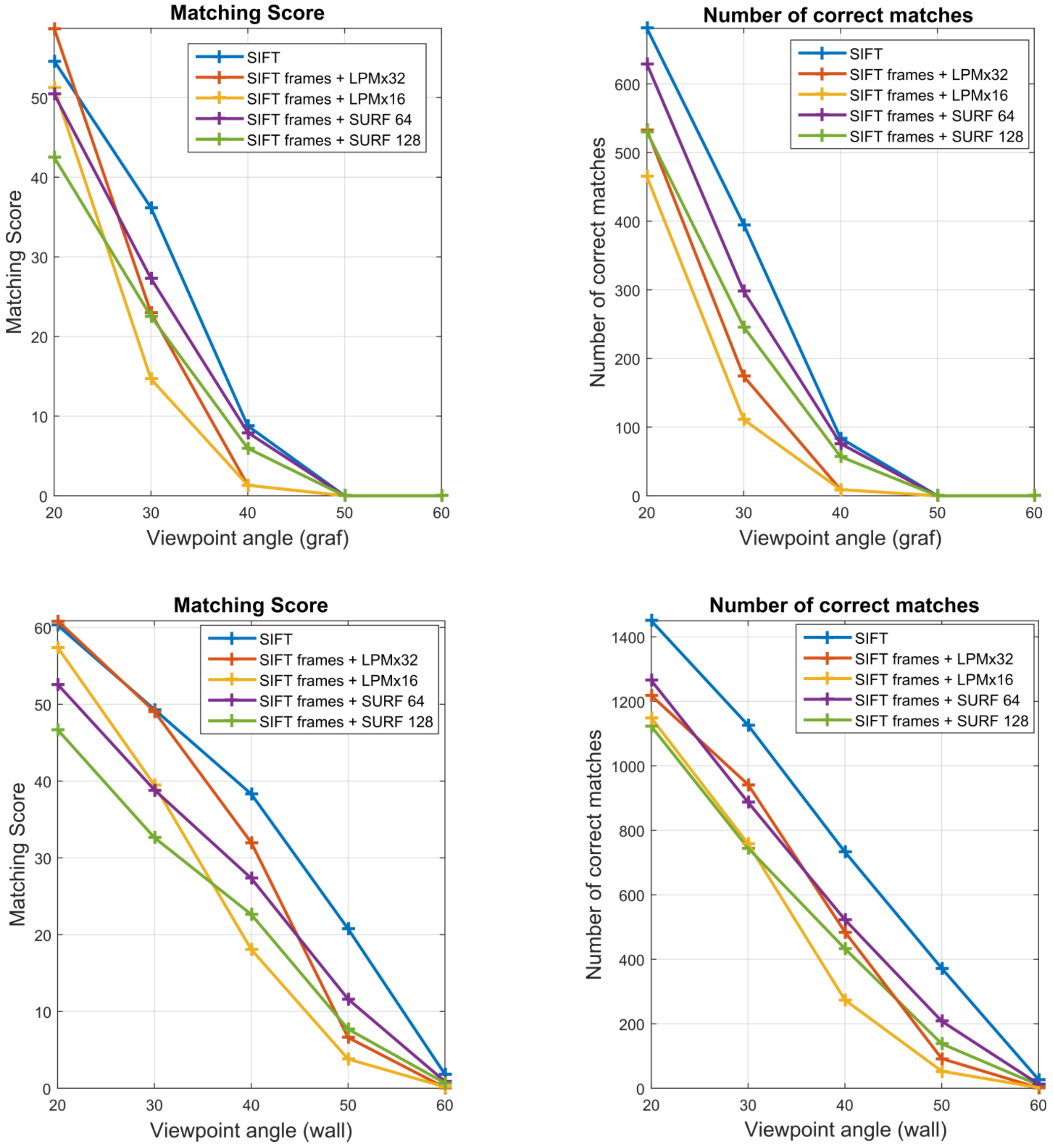

Figure 9: The VL benchmark performance comparison of the three descriptors: SIFT, LPM (in the two sampling variants: 16 x 16 and 32 x 32 resulting in 48- and 56-long feature vectors respectively), and SURF (with the two feature vector lengths: 64 and 128). The same feature detector (SIFT) was used with all descriptors. The plots present results for the two image subsets with different viewpoint angles in the Oxford dataset: *graf* (upper row) and *wall* (lower row).

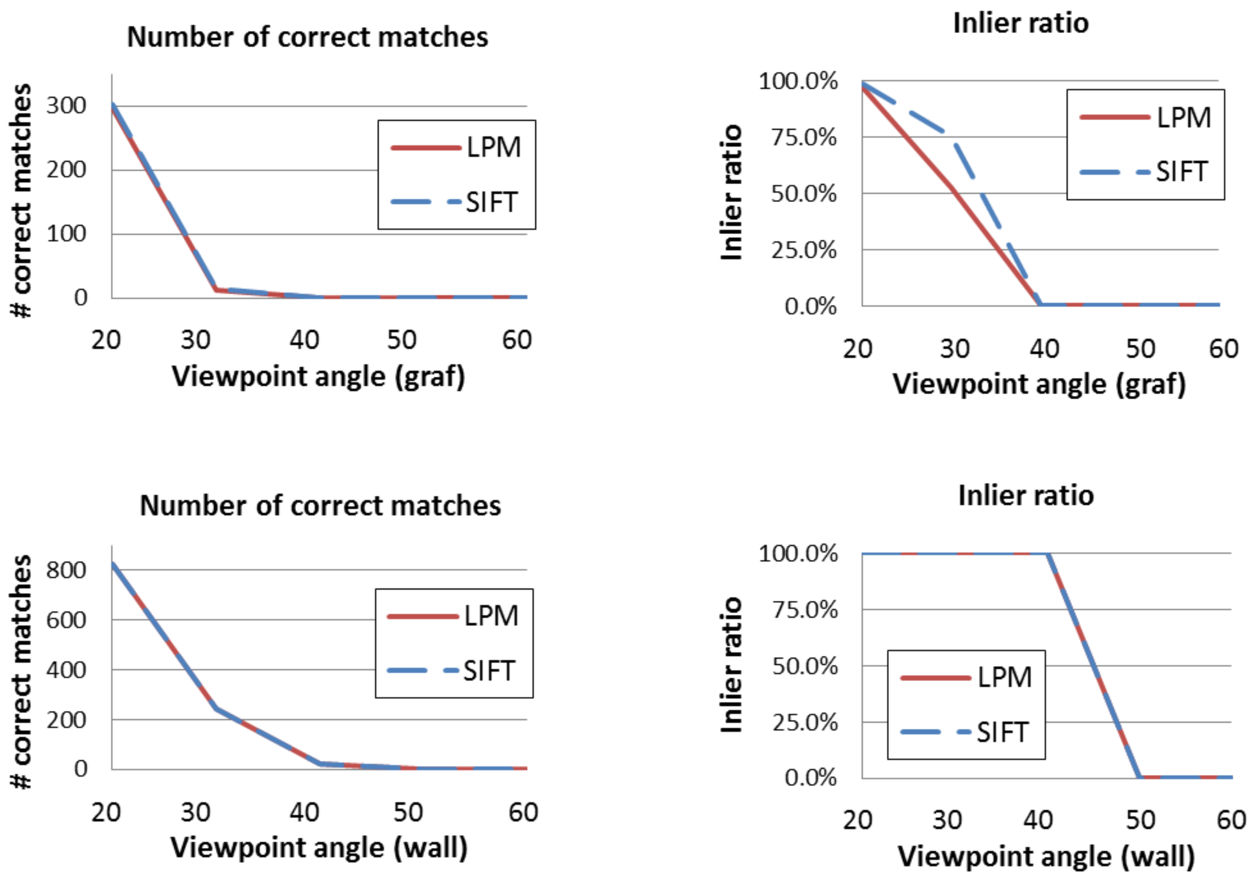

Figure 10: Performance comparison between SIFT and LPM with the 32 x 32 sampling and 56-long feature vectors. The same feature detector (SIFT) was used with both descriptors. The plots present the results of the alternative (threshold- and RANSAC-based) evaluation framework for the two image subsets with different viewpoint angles in the Oxford dataset: *graf* (upper row) and *wall* (lower row).

Evaluation – Transmission Electron Microscopy

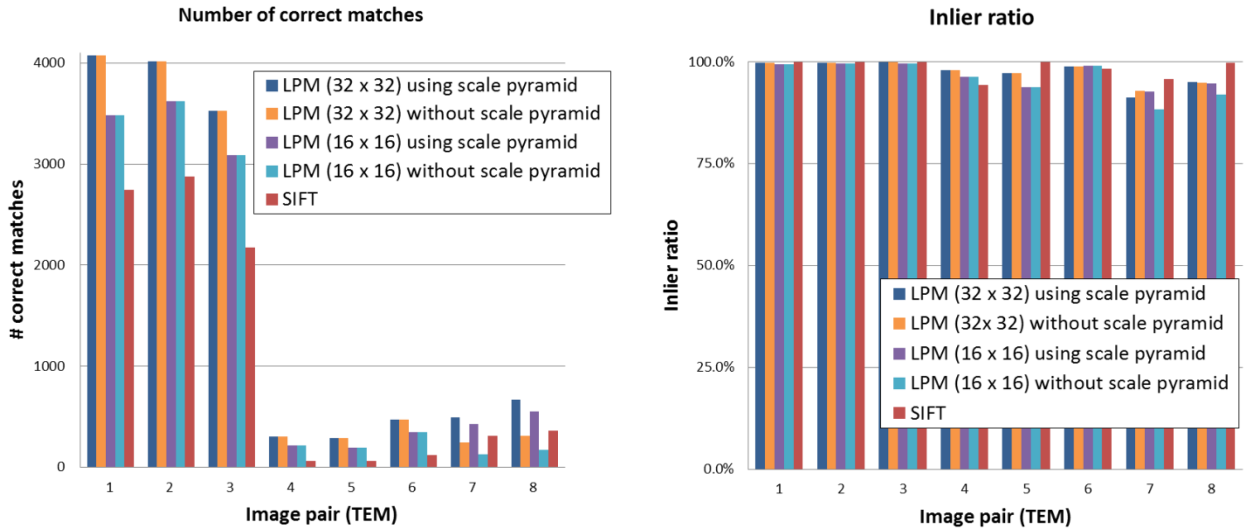

Figure 11: Comparison of the feature matching results obtained with SIFT and the two Log Polar Magnitude descriptor (LPM) sampling versions on the features found with the SIFT detector. LPM was additionally tested with and without the scale pyramid. The bar charts present results for the transmission electron microscopy (TEM) image dataset. The first 6 image pairs contained only translation problems whereas the last two image pairs differed in the magnification levels.

On the last two images in this dataset, with large scale variations, the benefit of using the optional scale pyramid described in the Conclusion section is clearly demonstrated. Figure 11 shows results for SIFT and LPM in the two sampling variants with and without the scale pyramid. Obviously, when there is no scale difference between images (image pairs 1 to 6) using the scale pyramid gives exactly the same results as without it. However, when the scale difference is present between the images adding the scale pyramid analysis can substantially increase the number of correct matches found with LPM and make it outperform SIFT. However, the same approach could be used with SIFT which makes the comparison in this case unfair. Therefore, in all the previous experiments (including those which results are published in the Supplementary Information) we did not use the scale pyramid with any of the descriptors. If we consider only the results of LPM without the scale pyramid we can observe that its both sampling variants performed better than SIFT in the first 6 image pairs (those with translation only). In the case of the last two image pairs (those with different magnification) SIFT found more correct matches and achieved higher inlier ratio. Nevertheless, LPM with the 32 x 32 sampling and no pyramid scale was only slightly worse than SIFT in the number of correct matches.

Computational complexity

LPM descriptor

Any descriptor is obviously linear with respect to the number of detected image features. Hence, we are analyzing the LPM complexity for a single image feature. The Log Polar Transform has the complexity

$$LPT = O(N\,M\,G^2)$$

where N x M is the sampling resolution and G is the size of the Gaussian sampling. The complexity of the 1D FFT is

$$FFT_{1D} = O(k\,N\log N)$$

where N is the number of samples and k is a constant. Hence, the complexity of the 2D FFT is

$$FFT_{2D} = O(N\,k\,M\log M + M\,k\,N\log N) = O\big(k\,M\,N\,(\log M + \log N)\big) = 0(k\,M\,N\log(M\,N))$$

$$FFT_{2D} = O(N\,M\log(N\,M))$$

Calculating the magnitude of the Fourier spectrum is

$$|F| = O(N\,M)$$

Finally, the feature selection is linear with respect to the feature vector length L, $O(L)$. Therefore, the LPM complexity is

$$LPM = O(N\,M\,G^2 + N\,M\log(N\,M) + N\,M + L)$$

Assuming $N = M$

$$LPM = O(N^2\,G^2 + N^2\log(N^2) + N^2 + L)$$

$G < N$, moreover, G and L are constants that do not affect much the computation complexity, hence, it can be simplified to

$$LPM = O(N^2 + N^2 2\log(N) + N^2) = O(N^2\log N)$$

LPM has the same complexity as its slowest component – FFT.

Feature matching

In case of the exhaustive matching of X and Y feature vectors of length L the complexity is

$$Matching = O(X\ Y\ L)$$

The matching is linear with respect to the feature vector length, and hence, a shorter feature vector will result in a faster matching. Note that matching floating point vectors is of course slower than matching integers. However, it is a common practice to normalize the feature vectors to a unit length (which turns all values to floating points) before the matching and hence, what really matters is the feature vector length.
